# Analytical evaluation of three enzymatic assays for measuring total bile acids in plasma using a fully-automated clinical chemistry platform

**DOI:** 10.1371/journal.pone.0179200

**Published:** 2017-06-08

**Authors:** Elisa Danese, Gian Luca Salvagno, Davide Negrini, Giorgio Brocco, Martina Montagnana, Giuseppe Lippi

**Affiliations:** Section of Clinical Biochemistry, University of Verona, Verona, Italy; University of Cambridge, UNITED KINGDOM

## Abstract

**Background:**

Although the clinical significance of measuring bile acids concentration in plasma or serum has been recognized for long in patients with hepatobiliary disease and/or bile acid malabsorption, the reference separation techniques are expensive and mostly unsuitable for early diagnosis and for measuring large volumes of samples. Therefore, this study was aimed to evaluate the analytical performance of three commercial enzymatic techniques for measuring total bile acids in plasma using a fully-automated clinical chemistry platform.

**Methods:**

Three commercial enzymatic assays (from Diazyme, Randox and Sentinel) were adapted for use on a Cobas Roche c501. We performed imprecision and linearity studies, and we compared results with those obtained using a reference liquid chromatography-mass spectrometry (LC-MS) technique on an identical set of lithium-heparin plasma samples.

**Results:**

Total imprecision was optimal, always equal or lower than 3%. All assays had optimal linearity between 3–138 μmol/L. The comparison studies showed good correlation with LC-MS data (Spearman’s correlation coefficients always >0.92), but all plasma samples values were significantly underestimated using the commercial enzymatic assays (-44% for Diazyme, -16% for Randox and -12% for Sentinel). The agreement at the 10 and 40 μmol/L diagnostic thresholds of total bile acids in plasma ranged between 86–92%. This discrepancy was found to be mainly attributable to a heterogeneous composition in terms of bile acids content of the three assay calibrators.

**Conclusions:**

This study suggests that the analytical performance of the three commercial enzymatic assays is excellent, thus confirming that automation of this important test by means of enzymatic assessment may be feasible, practical, reliable and supposedly cheap. Nevertheless, the underestimation of values compared to the reference LC-MS also suggests that the local definition and validation of reference ranges according to the combination between the specific enzymatic assay and the different clinical chemistry platforms may be advisable.

## Introduction

The bile acids, 24-carbon steroids synthesized by the liver from a cholesterol molecule, are predominantly found in the bile of mammals and other vertebrates. Bile acids are conventionally divided in primary (i.e., cholic acid and [CA] chenodeoxyclolic acid [CDCA], directly synthesized in the liver) and secondary (i.e., deoxycolic acid [DCA], lithocholic acid [LCA] and ursodeoxycholic acid [UDCA], originating in the intestine from bacterial metabolism of the two primary bile acids). In humans, CA is also rapidly conjugated with glycine or taurine and hence converted in taurocholic acid (TCA) and glycocholic acid (GCA), whereas CDCA is conjugated with glycine or taurine and hence converted into taurochenodeoxycholic acid (TCDCA) and glycochenodeoxycholic acid (GCDCA), which are present in the bile at roughly identical concentration [1]. Human bile acids mostly consist (over 90%) of DCA and the conjugates of both cholic and chenodeoxyclolic acids. Importantly, the production of bile acids is a major source of cholesterol metabolism in humans. In humans, the physiologic secretion of bile acids varies between 12 and 18 g per day, but approximately 95% of this pool is actively reabsorbed in the ileum and recycled by the liver to be further secreted into the biliary system and gallbladder, so allowing to maintain a modest rate of liver synthesis compounded by a large recycle process and intestine secretion.

Recent evidence suggests that bile acids may exert additional pleotropic functions, including the regulation of glucose metabolism, control of signaling events in liver regeneration and regulation of overall energy expenditure [2,3]. Due to their primary role in bile metabolism, the physiological concentration of bile acids in blood is low, usually comprised between 2 and 10 μmol/L. Nevertheless, the blood concentration can dramatically increase, occasionally over 180 μmol/L, in many disease conditions such as biliary obstruction, biliary fistula, intrahepatic cholestasis, cholecystectomy, ileal resection, portal systemic venous shunting, acute hepatitis, chronic hepatitis, liver sclerosis and liver cancer [1]. Genetic abnormalities in biosynthesis of bile acids have also been described. [1].

The clinical value of measuring bile acids concentration in blood has been recognized for long in evaluating patients with hepatobiliary disease and/or bile acid malabsorption [4,5]. The longitudinal assessment of total bile acids concentration has also been proposed for monitoring enterohepatic circulation of bile acids and liver transport capacity. Notably, the interest for measuring total bile acid in serum or plasma for diagnosing intrahepatic cholestasis in pregnancy has been recently reaffirmed and endorsed by a number of scientific societies [6–9]. In particular, the guidelines of the UK Royal College of Obstetricians and Gynaecologists states that abnormal values of aminotransferases, gamma-glutamyl transferase (GGT) and total bile acids are sufficient to support the diagnosis of obstetric cholestasis [9], and also provide valuable therapeutic information for preventing foetal complications attributable to the toxic effects of bile salts [10]. The reference range of total bile acids in the serum of pregnant women has been recently established at 0.3–10 μmol/L [11], whereas serum or plasma values >40 μmol/L are diagnostics of severe obstetric cholestasis and were found to be strongly associated with impaired foetal outcome [12].

Despite emerging evidence that the assessment of total bile acids in blood may provide valuable clinical information over a broad spectrum of liver disease, their analysis remains challenging due to the complex nature of the molecules and the relatively low concentration in common biological fluids such as blood, serum or plasma [13]. A number of techniques have been proposed for bile acids measurements over the past decades, including thin-layer chromatography, gas chromatography, high-performance liquid chromatography (HPLC), liquid chromatography-mass spectrometry (LC-MS), gas chromatography-mass spectrometry (GC-MS) supercritical fluid chromatography and capillary electrophoresis, along with immunoassays and bioluminescence assays [14]. Separation techniques such as LC-MS and GC-MS remain the gold standards for obtaining accurate measurements of these steroidal molecules, but their widespread applicability in clinical laboratories is strongly limited by the need of purchasing dedicated and expensive instrumentation, along with the long turnaround time which makes these methods almost unsuitable for fast diagnosis in emergency settings. Notably, an alternative and easier approach has been proposed, entailing the enzymatic assessment of total bile acids, without prior extraction, and based on the use of 3-alpha-hydroxysteroid dehydrogenase (i.e., the so-called “cycling method”) [15–17]. Therefore, the aim of this article was to evaluate the analytical performance of three commercial enzymatic techniques for measuring total bile acids in plasma using a fully-automated clinical chemistry platform and to compare results with those obtained using the reference LC-MS technique.

## Materials and methods

### Methods description

Three enzymatic assays for the measurement of total bile acids in plasma were adapted for use on a fully-automated clinical chemistry analyzer (Roche Cobas 501; Roche Diagnostics GmbH, Penzberg, Germany), according to manufacturers’ protocols.

The Diazyme total bile acids assay (Diazyme Europe GmbH, Dresden, Deutschland) is an enzymatic colorimetric technique for quantitative assessment of total bile acids in serum, EDTA or heparinized plasma. Briefly, in the presence of Thio-NAD, the enzyme 3-α-hydroxysteroid dehydrogenase (3-α-HSD) converts bile acids to 3-keto steroids and Thio-NADH. The reaction is reversible and 3-α-HSD can convert 3-keto steroids and Thio-NADH to bile acids and Thio-NAD. In the presence of excess NADH, the enzyme cycling occurs efficiently and the rate of formation of Thio-NADH is assessed by measuring specific change of absorbance at 405 nm. The linearity, analytical sensitivity and the upper limit of the reference range of this technique, as quoted by the manufacturer, are 0–180 μmol/L, 1 μmol/L and 10 μmol/L, respectively. The sample volume needed for the assay is 4 μL.

The Randox total bile acids assay is an enzymatic colorimetric technique for quantitative assessment of total bile acids in serum, EDTA or heparinized plasma. Briefly, two reactions are combined in this kinetic enzyme cycling method. In the former reaction, bile acids are oxidised by 3-α-HSD with the subsequent reduction of Thio-NAD to Thio-NADH. In the latter reaction, the oxidised bile acids are reduced by the same enzyme with the subsequent oxidation of NADH to NAD. The rate of formation of Thio-NADH is assessed by measuring specific absorbance change at 405 nm. The linearity, analytical sensitivity and the upper limit of the reference range, as quoted by the manufacturer, are 0–188 μmol/L, 3.2 μmol/L and 10 μmol/L, respectively. The sample volume needed for the assay is 15 μL.

The Sentinel total bile acids assay is another enzymatic colorimetric assay for quantitative assessment of total bile acids in serum or plasma. Briefly, the enzyme 3-α-HSD converts bile acids to 3-ketosteroids and NADH in the presence of NAD. The NADH formed reacts with nitrotetrazolium blue (NBT) to form a formazan dye in the presence of diaphorase enzyme. The colour intensity of the formazan dye is proportional to bile acids concentration in the sample. The linearity, analytical sensitivity and the upper limit of the reference range of this technique, as quoted by the manufacturer, are 1–200 μmol/L, 1 μmol/L and 10 μmol/L, respectively. The sample volume needed for the assay is 200 μL.

The reference technique for assessing bile acids in this study entailed the use of a LC-MS assay, with separate detection of most representative bile acids in serum or plasma (i.e., representing over 99% of total bile acids contents in human blood) [18]: CA, DCA, CDCA, UDCA, TCDCA, GCA, GCDCA, glycoursodeoxycholic acid (GUDCA), hyodeoxycholic acid (HDCA), glycodeoxycholic acid (GDCA), LCA and TCA. Three deuterated internal standards (IS) were used for quantification: cholic-2,2,4,4-d4 Acid (CA-d4), deoxycholic-2,2,4,4-d4 acid (DCA-d4), chenodeoxycholic -2,2,4,4-d4 acid (CDCA-d4). All reagents, including UHPLC-grade methanol, acetonitrile and ammonium formate, were purchased from Sigma Aldrich (St. Louis, MO, USA) or Santa Cruz (CA, USA). Chromatographic separation was performed using Nexera X2 series UHPLC (Shimadzu, Kyoto, Japan) equipped with a Phenomenex Kinetex C18 (500 x 2.1 mm, 2.6 μm) column. The column temperature was 50°C. The mobile phase A consisted of 5 mM aqueous ammonium formate; the mobile phase B consisted of acetonitrile and methanol (50:50). A gradient elution with a flow rate of 0.5 mL/min was performed with 30% B for 0.3 min, a linear increase to 90% B until 5 min, followed by 90% B from 5 until 7 min and re-equilibration from 7.1 to 12 min with 30% B. An example of the chromatographic separation of the 12 BAs (calibration standard) is shown in S1 Fig.

The UHPLC system was coupled to a 4500 MD triple quadrupole mass spectrometer (AB Sciex, Darmstadt, Germany). Electrospray ionization was performed in the negative mode using the following parameters: 30 psi (curtain gas), 550°C (Source Temperature) -3500 V (Ion Spray Voltage), 45 psi/55 psi (Ion Gas 1 and 2, respectively). Data were recorded in the multiple reaction monitoring mode (MRM) with nitrogen as a collision gas. System operation, data acquisition and subsequent quantification were achieved by using Analyst 1.6.2. software (AB Sciex) and MultiQuant 3.0.2. (AB Sciex). Declustering potential, collision cell parameters and transitions were optimized for each compound. The 12 BAs were assessed against a suitable d4-BA chosen on the basis of structural similarity (S1 Table).

Mixed stock solution containing each bile acid standard was prepared in methanol and then further diluted with methanol:water (50:50, v:v) to obtain a 6 points calibration curve. The sample preparation procedure was based on previously published methods [19,20] with slight modifications. Briefly, 500 μL of lithium-heparin plasma samples were spiked with 5μL of IS-mixture (24 μmol/L D4-CA, 25μmol/L D4-DCA, 25 μmol/L D4-CDCA), mixed with 1000 μL of acetonitrile and then vortexed for 1 min. After 15 min centrifugation at 13000 g, 500 μL of the supernatant were transferred to the autosampler vials and used for UHPL-MS analysis. The injection volume was 2 μL. The limit of quantitation (LOQ), determined as a signal-to-noise ratio of ten, was below 20 nmol/L for all bile acid species. The intra-assay imprecision was comprised between 1.2–2.4%, whereas the inter-assay imprecision was comprised between 4.4–6.4%. The data of instrument setting are shown in S1 Table. The total concentration of bile acids in plasm was finally obtained by summing data obtained from the single measurement of each of the 12 bile acids.

The study was carried out in accordance with the Declaration of Helsinki, was approved by the local ethical committee (University Hospital of Verona Institutional Review Board) and was performed under the terms of all relevant local legislation. No consent was required as the study design implies that the data were analyzed anonymously.

### Imprecision studies

The imprecision studies for the three commercial enzymatic assays were carried out using two plasma pools, displaying low (~16 μmol/L) and high (~135 μmol/L) total bile acids concentration. Each plasma pool was obtained by pooling 25 anonymized plasma samples referred to the local laboratory for routine testing and collected in evacuated blood tubes (Vacutest 3.5 mL, 75×13 mm, containing lithium-heparin; Kima, Padova, Italy). The plasma pools were then divided in 11 aliquots of 2.5 mL each, which were stored at -70°C until measurement. The intra-assay imprecision was evaluated by 15 sequential measurements of one aliquot of the two plasma pools, whereas the inter-assay imprecision was assessed by one measurement in ten consecutive working days of the two plasma pools. Final results were reported as coefficient of variation (CV).

### Linearity

An anonymized routine lithium-heparin plasma sample with high total bile acids value (~138 μmol/L) was serially diluted at fixed ratios (1:9; 2:8; 3:7; 4:6; 5:5; 6:4; 7:3, 8:2; 9:1) with an anonymized routine lithium-heparin plasma sample with very low total bile acids value (~3 μmol/L), to cover the most clinically significant range of concentrations of total bile acids in human plasma. Serial dilutions were analysed in duplicate with each of the three commercial enzymatic assays and the theoretical values were calculated from measured values of undiluted specimens. Linearity was assessed with calculation of Passing and Bablok regression and Spearman’s correlation coefficient (r).

### Comparison studies

The comparison studies were performed using 51 routine random and anonymized lithium-heparin plasma samples referred to the laboratory for routine testing. The results of the three enzymatic assays were compared with those obtained using the reference LC-MS technique, by Wilcoxon’s signed rank test and Spearman’s correlation. The mean percentage bias (and the 95% Confidence Interval; 95% CI) compared to the reference LC-MS was estimated using Bland-Altman plot analysis. The agreement (with kappa statistics) of values obtained using the three enzymatic assays compared to those obtained with the reference LC-MS technique was evaluated at the diagnostic thresholds of 10 μmol/L (i.e., the upper limit of the reference range) and 40 μmol/L (i.e., the cut-off for severe obstetric cholestasis) [11].

### Statistics

Results were shown as mean±standard deviation (SD). The statistical evaluation was performed with Analyse-it for Microsoft Excel (Analyse-it Software Ltd, Leeds, UK).

## Results

### Imprecision studies

The results of the imprecision studies using the three commercial enzymatic assays are shown in Table 1. The intra-assay imprecision was comprised between 0.44–1.66% for the plasma pool with low concentration of bile acids and between 0.84–2.18% for the plasma pool with high concentration of bile acids, respectively. The inter-assay imprecision was instead comprised between 2.47–3.91% for the plasma pool with low concentration of bile acids and between 1.02–2.37% for the plasma pool with high concentration of bile acids, respectively. The total analytical imprecision, calculated according to Krouwer and Rablnowitz [21] was found to be 2.84% for Diazyme, 3.02% for Randox and 2.26% for Sentinel, respectively.

**Table 1:** Analytical imprecision of three enzymatic techniques for measuring total bile acids.

	Intra-assay			Inter-assay		
	Mean (μmol/L)	SD (μmol/L)	CV	Mean (μmol/L)	SD (μmol/L)	CV
**Pool Low**						
Diazyme	16.60	0.07	0.44%	16.28	0.55	3.37%
Randox	14.68	0.16	1.12%	15.34	0.60	3.91%
Sentinel	18.35	0.31	1.66%	17.51	0.43	2.47%
**Pool High**						
Diazyme	118.56	2.59	2.18%	118.69	2.40	2.03%
Randox	137.03	1.33	0.97%	146.03	3.47	2.37%
Sentinel	150.31	1.26	0.84%	149.65	1.52	1.02%

SD, standard deviation; CV, coefficient of variation.

### Linearity studies

**Fig 1:**
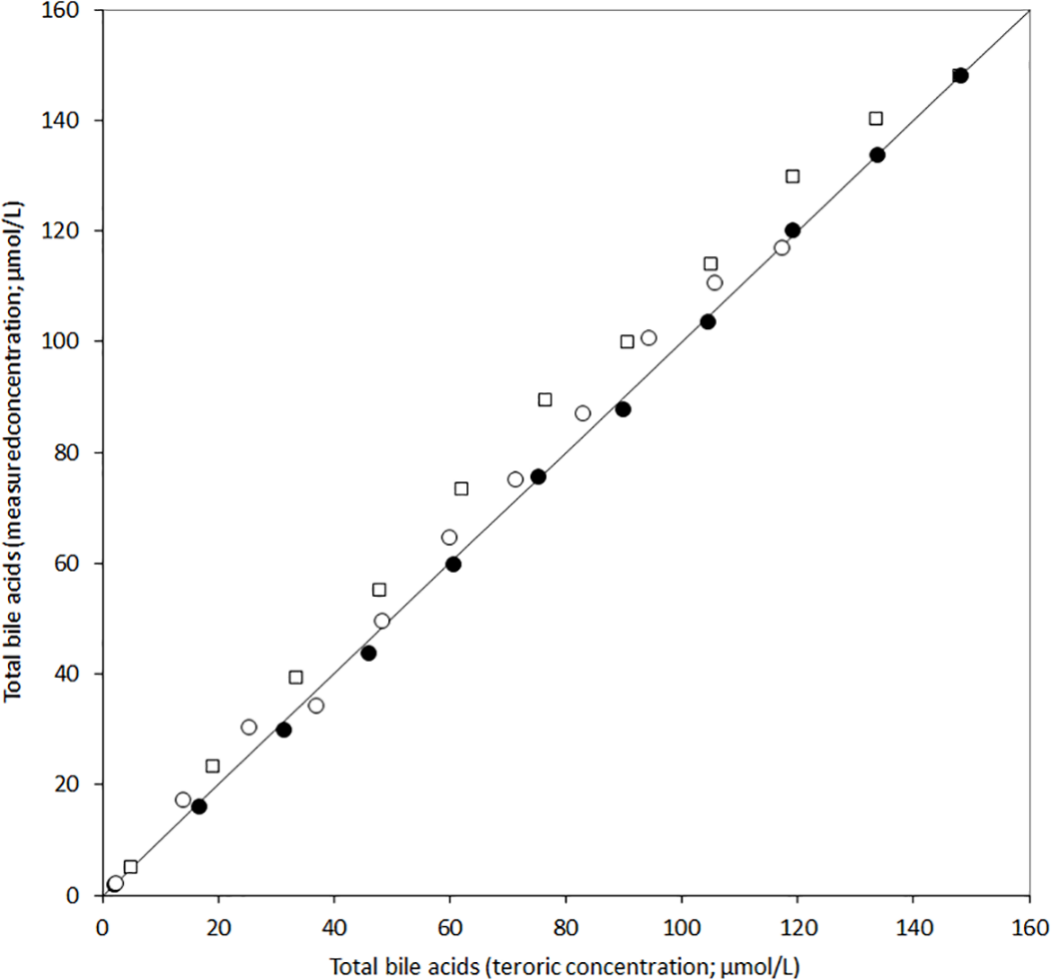
Results of the linearity studies of three enzymatic techniques for measuring total bile acids. Diazyme (○), Randox (●) and Sentinel (□).

The results of the linearity studies are shown in Fig 1. All the assays displayed excellent performance in the range of concentration between 3–138 μmol/L. More specifically, the coefficients of the Passing and Bablok regression analysis were *y* = 1.03*x* + 2.03 for Diazyme, *y* = 1.01*x* – 0.98 for Randox and *y* = 1.04*x* + 4.22 for Sentinel, respectively. The relative correlation coefficients were 0.998 (p<0.001) for Diazyme, 1.000 (p<0.001) for Randox and 0.996 (p<0.001) for Sentinel, respectively.

### Comparison studies

**Fig 2:**
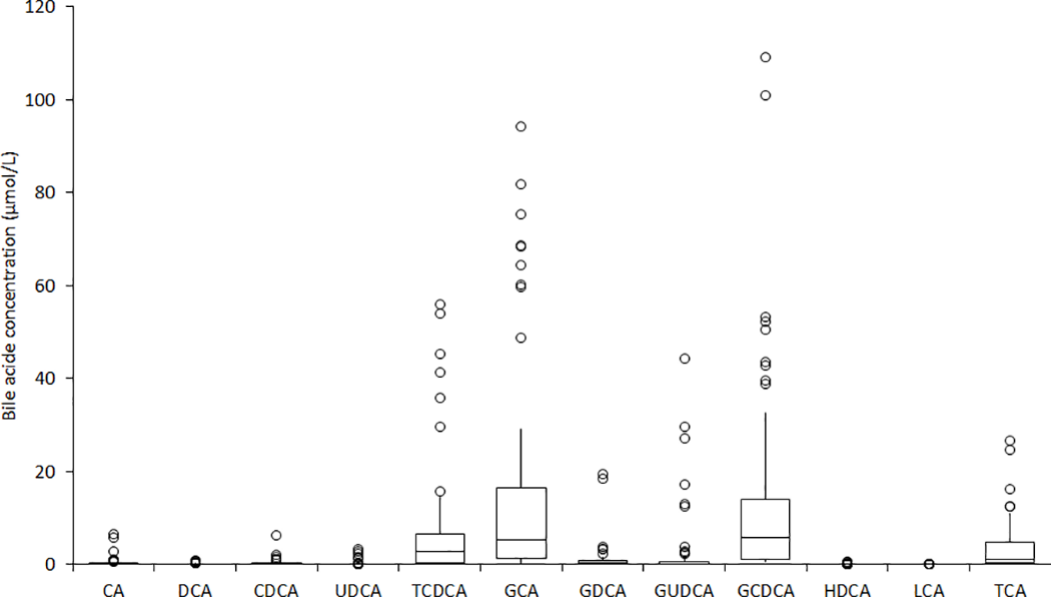
Bile acids composition of the 51 lithium-heparin plasma samples used for the comparison study. CA, cholic acid; DCA, deoxycholic acid; CDCA, chenodeoxycholic acid; UDCA, ursodeoxycholic acid; TCDCA, taurochenodeoxycholic acid; GA, glycocholic acid; GCDCA, glycochenodeoxycholic acid; GUDCA, glycoursodeoxycholic acid; HDCA, hyodeoxycholic acid; GDCA, glycodeoxycholic acid; LCA, lithocholic acid; TCA, taurocholic acid (TCA).

The data of the bile acids composition measured by LC-MS in 51 routine lithium-heparin plasma samples is shown in Fig 2. As predictable the highest plasma concentration was found for GCA (mean, 34%), followed by GCDCA (man, 28%), TCDCA (14%) and TCA (10%). Overall, these four molecules represented 85% of the total bile acids concentration measured in our lithium-heparin plasma samples. This finding is in accord with information provided in a separate study including 63 women with intrahepatic cholestasis of pregnancy and 26 normal pregnant women [22].

The three enzymatic assays were compared with the reference LC-MS technique using Spearman’s correlation, and the results are shown it Table 2. Overall, the Spearman’s correlation coefficient (r) was always >0.92 compared to LC-MS, and also higher than 0.89 comparing data obtained with the three different commercial assays (Table 2). The median (and interquartile range) of values of bile acids in plasma was 16.2 μmol/L (48.9 μmol/L) for LC-MS, 12.3 μmol/L (22.7 μmol/L) for Diazyme, 15.6 μmol/L (36.6 μmol/L) for Randox and 14.4 μmol/L (42.6 μmol/L) for Sentinel, respectively. In all cases the difference of values obtained with LC-MS and the three enzymatic assays was found to be statistically significant (all p<0.001).

**Table 2:** Spearman’s correlation for the measurement of total bile acids using three commercial kits and the reference liquid chromatography-mass spectrometry (LC-MS) assay.

	LC-MS	Randox	Sentinel
Diazyme	r = 0.92 (p<0.001)	r = 0.92 (p<0.001)	r = 0.89 (p<0.001)
Randox	r = 0.99 (p<0.001)	-	r = 0.97 (p<0.001)
Sentinel	r = 0.97 (p<0.001)	-	-

**Fig 3:**
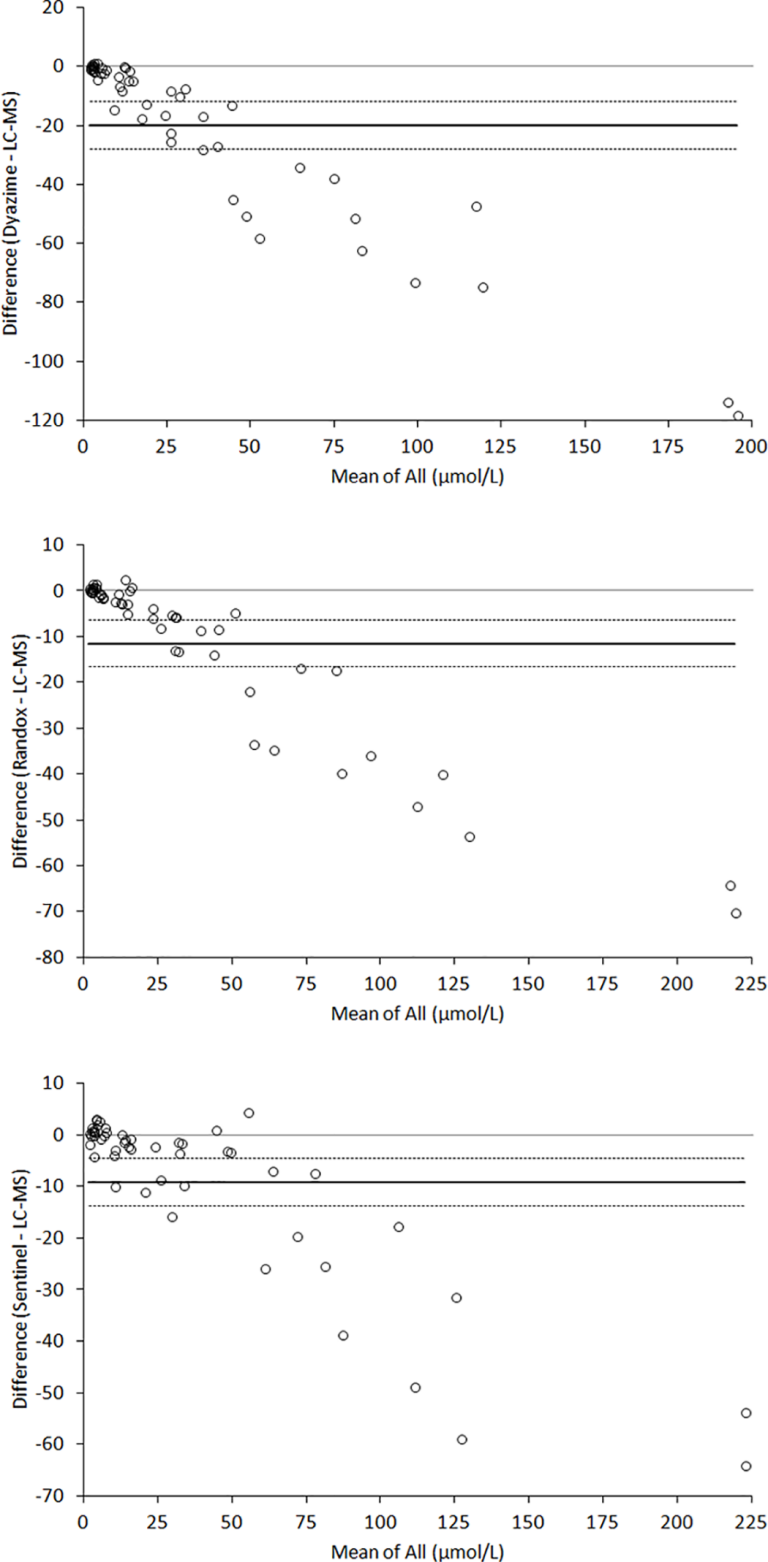
Bland and Altman plot analysis of three enzymatic techniques for measuring total bile acids in plasma compared to the reference liquid chromatography-mass spectrometry (LC-MS) assay. The horizontal lines are drawn at mean (continuous line) and 95% confidence interval (95% CI; dotted lines) of bias.

The mean absolute biases compared to the reference LC-MS technique was -20 μmol/L (95% CI, -28 to -12 μmol/L) for Diazyme, -11 μmol/L (95% CI, -17 to -6 μmol/L) for Randox and -9 μmol/L (95% CI, -14 to -4 μmol/L) for Sentinel, respectively (Fig 3). Accordingly, the mean percentage biases compared to the reference LC-MS technique was -44% (95% CI, -56 to -32%) for Diazyme, -16% (95% CI, -23 to -9%) for Randox and -12% (95% CI, -23% to -1%) for Sentinel. Interestingly, the negative bias versus the reference LC-MS technique was especially evident in lithium-heparin plasma samples with total bile acids concentration >10 μmol/L (n = 33; bias -60% and 95% CI -73 to -48% for Diazyme; bias -26% and 95% CI -32 to -20% for Randox; bias -23% and 95% CI -31 to -16% for Sentinel, respectively).

The agreement with LC-MS measurements at the 10 μmol/L threshold was 90% (kappa statistics, 0.79; 95% CI, 0.63–0.96) for Diazyme, 96% for Randox (kappa statistics, 0.92; 95% CI, 0.80–1.00) and 92% for Sentinel (kappa statistics, 0.83; 95% CI, 0.68–0.99), whereas the agreement at the 40 μmol/L threshold was 86% for Diazyme (kappa statistics, 0.64; 95% CI, 0.40–0.87), 96% (kappa statistics, 0.90; 95% CI, 0.77–1.03) for Randox and 100% for Sentinel (kappa statistics, 1.0).

Notably, the agreement at the 10 μmol/L threshold was 92% (kappa statistics, 0.84; 95% CI, 0.69–0.99) between Diazyme and Randox, 92% (kappa statistics, 0.84; 95% CI, 0.69–0.99) between Diazyme and Sentinel, 96% (kappa statistics, 0.92; 95% CI, 0.80–1.00) between Randox and Sentinel, whereas the agreement at the 40 μmol/L threshold was 90% (kappa statistics, 0.72; 95% CI, 0.50–0.94) between Diazyme and Randox, 86% (kappa statistics, 0.64; 95% CI, 0.40–0.87) between Diazyme and Sentinel, 96% (kappa statistics, 0.90; 95% CI, 0.77–1.00) between Randox and Sentinel, respectively.

### LC-MS analysis of the calibrators

**Fig 4:**
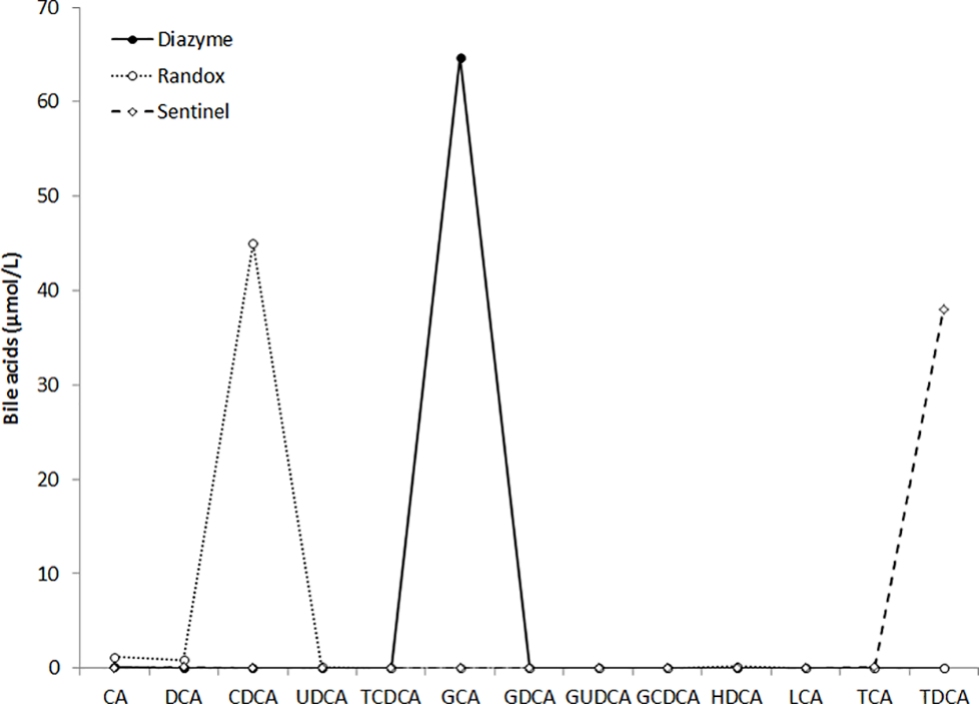
Bile acids composition of calibrators of the three commercial assays. CA, cholic acid; DCA, deoxycholic acid; CDCA, chenodeoxycholic acid; UDCA, ursodeoxycholic acid; TCDCA, taurochenodeoxycholic acid; GA, glycocholic acid; GCDCA, glycochenodeoxycholic acid; GUDCA, glycoursodeoxycholic acid; HDCA, hyodeoxycholic acid; GDCA, glycodeoxycholic acid; LCA, lithocholic acid; TCA, taurocholic acid; TDCA, taurodeoxycholic acid.

In order to define whether the differences of values observed with the three commercial enzymatic assays may be attributable to calibration, the calibrators of the three enzymatic kits were also subjected to analysis with LC-MS. The results of this test are shown in Fig 4, attesting that the three calibrators largely differ in terms of the bile acid concentration. More specifically, the Diazyme calibrator contains almost exclusively GCA, the Randox calibrator contains almost exclusively CDCA, whereas the Sentinel calibrator almost exclusively contains an additional bile acid other than those previously assessed, which was apparently identified as being taurodeoxycholic (TDCA). In accord with results of the comparison study and with the exception of the Sentinel calibrator, the value of total bile acids measured by LC-MS was found to be consistently higher (from 10–20%) than that labelled on the calibrator vial by Diazyme and Randox.

## Discussion

The clinical significance of measuring total bile acids for diagnosing and monitoring liver diseases is now well established. In particular, the role of this test has been recently reinforced by guidelines indicating that this measure can be extremely useful for diagnosing intrahepatic cholestasis in pregnancy [6–9]. Pařízek et al also recently reported that steroid profiling using GC-MS displays up to 0.93 sensitivity and 0.94 specificity for diagnosing intrahepatic cholestasis in pregnancy [23].

Despite validated separation techniques, especially LC-MS and GC-MS, are still regarded as reference methods for measuring bile acids in serum or plasma, their introduction in clinical practice is strongly limited by many aspects, including the long preanalytical management for preparing the samples before analysis, the high cost of instrumentation, the need of skilled personnel for performing the test, the long turnaround time and the low throughput, which ultimately make these techniques unsuitable for routine assessment, especially in an urgent clinical setting. The recent development of commercial enzymatic assays for measuring total bile acids in serum or plasma should hence be considered a valuable perspective, since these assays are suitable for automation and can hence support the generation of a large number of test results in a short time. Nevertheless, as for any other laboratory test, a preliminary analytical validation is mandatory before these methods can be introduced into clinical and laboratory practice, especially using fully-automated clinical chemistry platforms.

The results of our study demonstrate that the automatization of three commercial enzymatic assays for total bile acids quantification may be a suitable and reliable alternative to LC-MS. The intra- and inter-assay imprecision of these kits was found to be particularly favourable, with CV always lower than 4%, thus enabling accurate diagnosis and monitoring of liver diseases (Table 1). The linearity of the different techniques was also excellent, up to 160 μmol/L, with CVs always greater than 0.996 (Fig 1).

The results of the comparison studies, using LC-MS as the reference technique, deserve a detailed discussion. The correlation of the three enzymatic methods with LC-MS data was excellent, always greater than 0.92 (Table 2). Nevertheless, the analysis of the Bland and Altman plot analysis suggests that all the commercial enzymatic assays substantially underestimate the value of total bile acids in plasma, with bias comprised between -12% to -44% (Fig 3). The bias was particularly evident in lithium-heparin plasma samples with total bile acids concentration >10 μmol/L, being comprised between -23% and -60%. This consistent bias is also reflected in the agreement at the two clinical thresholds of 10 and 40 μmol/L, which ranged overall between 86–100%, with the Sentinel enzymatic assays displaying the best overall agreement and the Diazyme enzymatic assay exhibiting the highest underestimation of values and the lowest agreement with the reference LC-MS technique. This is not surprising, since a previous study also showed that enzymatic assays display variable recovery of some species of bile acids (e.g., CA, CDCA and DCA), and some these kits generate a consistent underestimation of data compared to LC-MS [24]. This important information, confirmed in our investigation, highlights an important issue, that is the still insufficient standardization and harmonization of the different enzymatic assays, which could also be confirmed by our analysis of the calibrators of the three commercial kits using LC-MS (Fig 4).

Although the reference LC-MS assay has been used for estimating a large number of bile acids in our study, we did not assess the total bile acid pool in human plasma, so that the bias observed with the commercial enzymatic assays should be considered an overall approximation of the real bias. In conclusion, the results of this study, which is the first aimed to evaluate the analytical performance of three commercial enzymatic assays for measuring total bile acids concentration in plasma or serum to the best of our knowledge, have some strengths and also significant practical implications. Unlike a previous study, which used LC-MS as the reference technique but only measured a rather limited number of bile acids [24], we extended the panel of bile acids measurement up to twelve different compounds, so virtually covering the essential range of these molecules in human plasma and serum. Overall, the analytical performance of the different commercial kits was found to be excellent, thus confirming that automation of this important test by means of enzymatic assessment may be feasible, practical, reliable and supposedly cheap. These methods can hence be considered valid surrogates of LC-MS in clinical practice, so offering a valuable opportunity to clinical laboratories for performing rapid, accurate and high throughput analyses for both diagnosing and monitoring liver diseases. Nevertheless, the consistent underestimation observed comparing data using the three enzymatic assays with those obtained with LC-MS also suggests that harmonization of commercial assays is still a largely unmet target for this measurement and that additional recovery experiments may be required to address the current analytical bias This conclusion is strongly supported by LC-MS analysis of the assay calibrators (Fig 4), showing a heterogeneous composition in terms of bile acids contents as well as a substantial discrepancy between LC-MS measurements and labelled values of the individual calibrators. Therefore, local definition and validation of reference ranges according to the combination between the specific enzymatic assay and the different clinical chemistry platforms may be advisable for harmonizing data and increasing diagnostic accuracy at the clinically relevant thresholds of bile acids in serum or plasma, before the commercial enzymatic assays can be introduced into routine clinical practice.

## Supporting information

S1 Fig Chromatographic separation of 12 BAs (calibration standard). (TIF)

S1 Table Instrument setting. RT: retention time, DP: declustering potential, EP: entrance potential, CE: collision energy, CXP: collision cell exit potential, IS: internal standard.
(DOCX)
