# Supplementary figures and images for: Analytical evaluation of three enzymatic assays for measuring total bile acids in plasma using a fully-automated clinical chemistry platform
Source: PLoS One. 2017 Jun 8;12(6):e0179200. doi: 10.1371/journal.pone.0179200 (PMC5464614; doi:10.1371/journal.pone.0179200)

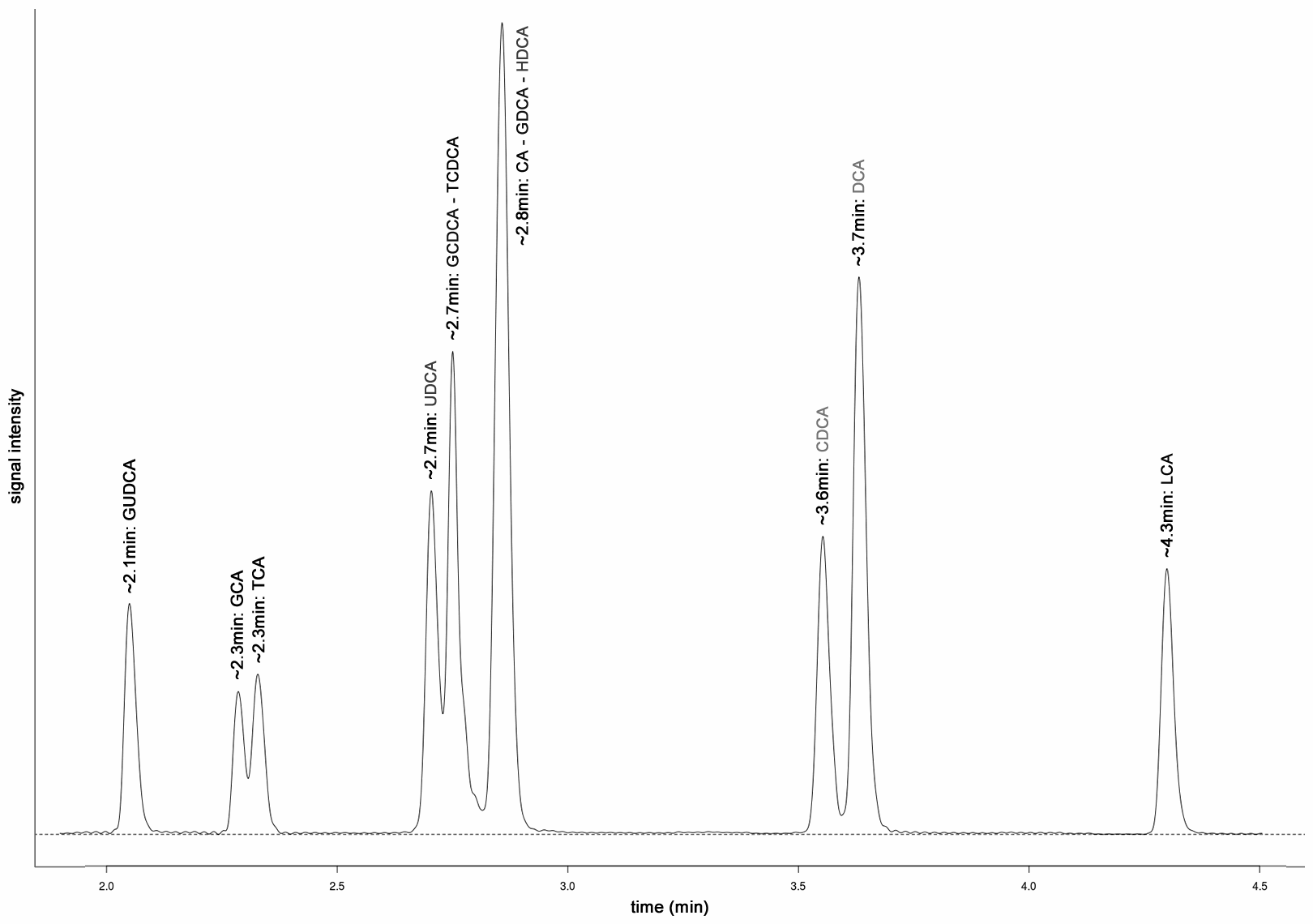

Supplement: S1 Fig — (TIF) [file pone.0179200.s001.tif]
