# Supplementary material for: Analytical evaluation of three enzymatic assays for measuring total bile acids in plasma using a fully-automated clinical chemistry platform
Source: PLoS One. 2017 Jun 8;12(6):e0179200. doi: 10.1371/journal.pone.0179200 (PMC5464614; doi:10.1371/journal.pone.0179200)
Supplement: S1 Table — RT: retention time, DP: declustering potential, EP: entrance potential, CE: collision energy, CXP: collision cell exit potential, IS: internal standard. (DOCX) [file pone.0179200.s002.docx]

**Table S1**: Instrument setting

| **ID** | **Q1 Mass (Da)** | **Q2 Mass (Da)** | **RT (min)** | **DP (V)** | **EP (V)** | **CE (V)** | **CXP (V)** | **IS** |
| --- | --- | --- | --- | --- | --- | --- | --- | --- |
| CA-1 | 407,1 | 407,1 | 2,94 | -125 | -10 | -15 | -15 | D4-CA |
| CA-2 | 407,1 | 343 | 2,94 | -125 | -10 | -46 | -15 | D4-CA |
| DCA-1 | 391,31 | 391,3 | 3,68 | -125 | -10 | -14 | -5 | D4-DCA |
| DCA-2 | 391,3 | 345,3 | 3,68 | -125 | -10 | -46 | -11 | D4-DCA |
| CDCA-1 | 391,3 | 391,3 | 3,61 | -125 | -10 | -14 | -5 | D4-CDCA |
| CDCA-2 | 437,1 | 391,3 | 3,61 | -35 | -10 | -14 | -13 | D4-CDCA |
| UDCA-1 | 391,2 | 391,2 | 2,79 | -130 | -10 | -20 | -13 | D4-DCA |
| UDCA-2 | 437,1 | 391,2 | 2,79 | -33 | -10 | -30 | -13 | D4-DCA |
| TCDCA-1 | 498,3 | 498,3 | 2,79 | -180 | -10 | -14 | -17 | D4-CDCA |
| TCDCA-2 | 498,3 | 80 | 2,79 | -180 | -10 | -105 | -10 | D4-CDCA |
| GCA-1 | 464,3 | 464,3 | 2,3 | -120 | -10 | -20 | -20 | D4-CA |
| GCA-2 | 464,3 | 74 | 2,3 | -120 | -10 | -80 | -7 | D4-CA |
| GDCA-1 | 448,31 | 448,3 | 2,87 | -120 | -10 | -14 | -13 | D4-DCA |
| GDCA-2 | 448,31 | 74 | 2,87 | -120 | -10 | -64 | -13 | D4-DCA |
| GUDCA-1 | 448,4 | 448,3 | 2,07 | -120 | -10 | -12 | -15 | D4-CA |
| GUDCA-2 | 448,4 | 74 | 2,07 | -120 | -10 | -64 | -7 | D4-CA |
| GCDCA-1 | 448,3 | 448,3 | 2,78 | -120 | -10 | -14 | -13 | D4-CDCA |
| GCDCA-2 | 448,3 | 74 | 2,78 | -120 | -10 | -64 | -13 | D4-CDCA |
| HDCA-1 | 391,21 | 391,2 | 2,93 | -130 | -10 | -20 | -13 | D4-DCA |
| HDCA -2 | 437,11 | 391,2 | 2,93 | -30 | -10 | -30 | -13 | D4-DCA |
| LCA-1 | 375,3 | 375,3 | 4,31 | -135 | -10 | -12 | -13 | D4-DCA |
| LCA-2 | 421,1 | 375,3 | 4,31 | -40 | -10 | -34 | -11 | D4-DCA |
| TCA-1 | 514,3 | 514,3 | 2,33 | -180 | -10 | -18 | -19 | D4-CA |
| TCA-2 | 514,3 | 80 | 2,33 | -180 | -10 | -105 | -10 | D4-CA |

RT: retention time, DP: declustering potential, EP: entrance potential, CE: collision energy, CXP: collision cell exit potential, IS: internal standard.
